# Molecular Dynamic Simulation of the Self-Assembly of DAP12-NKG2C Activating Immunoreceptor Complex

**DOI:** 10.1371/journal.pone.0105560

**Published:** 2014-08-22

**Authors:** Peng Wei, Lida Xu, Cheng-Dong Li, Fu-De Sun, Long Chen, Tianwei Tan, Shi-Zhong Luo

**Affiliations:** Beijing Key Laboratory of Bioprocess, College of Life Science and Technology, Beijing University of Chemical Technology, Beijing, P. R. China; Hungarian Academy of Sciences, Hungary

## Abstract

The DAP12-NKG2C activating immunoreceptor complex is one of the multisubunit transmembrane protein complexes in which ligand-binding receptor chains assemble with dimeric signal-transducing modules through non-covalent associations in their transmembrane (TM) domains. In this work, both coarse grained and atomistic molecular dynamic simulation methods were applied to investigate the self-assembly dynamics of the transmembrane domains of the DAP12-NKG2C activating immunoreceptor complex. Through simulating the dynamics of DAP12-NKG2C TM heterotrimer and point mutations, we demonstrated that a five-polar-residue motif including: 2 Asps and 2 Thrs in DAP12 dimer, as well as 1 Lys in NKG2C TM plays an important role in the assembly structure of the DAP12-NKG2C TM heterotrimer. Furthermore, we provided clear evidences to exclude the possibility that another NKG2C could stably associate with the DAP12-NKG2C heterotrimer. Based on the simulation results, we proposed a revised model for the self-assembly of DAP12-NKG2C activating immunoreceptor complex, along with a plausible explanation for the association of only one NKG2C with a DAP12 dimer.

## Introduction

Most activating immunoreceptor complexes are multisubunit-transmembrane protein complexes in which ligand-binding and signal-transducing functions are contributed by separate protein modules that assemble in the endoplasmic reticulum to form functional complexes [1]. A common feature of the aforementioned complexes is the absence of signaling modules in the cytoplasmic domains of the ligand-binding receptor chain, which assembles with dimeric signal-transducing modules with cytoplasmic immunoreceptor tyrosine-based activation motifs (ITAMs) through non-covalent associations in their transmembrane domains [2]. Among them, one of the important members is the signaling modules-DAP12 (DNAX activation protein of 12 kDa) related activating immunoreceptor complex [3]. DAP12 is a relatively short polypeptide of only 113–114 amino acids, consisting of a short extracellular tail, a single transmembrane domain and cytoplasmic ITAMs. It ordinarily forms a disulfide-bonded homodimer, and associates with the receptor-natural killer group 2C (NKG2C) through contacts between the basic and acidic residues of transmembrane domains within the context of the hydrophobic lipid bilayer [4]. NKG2C forms a heterodimer with the C-type lectin CD94 in natural killer (NK) cells and binds to the non-classical major histocompatibility complex (MHC) molecule HLA-E [5]–[7]. The resulting DAP12-NKG2C activating immunoreceptor complex delivers activating signals through the ITAM motifs in the DAP12 cytoplasmic tails, which is thought to have an important role in maintaining the balance of positive and negative signals governing NK cell activation and inhibition [8], [9]. The sophisticated repertoire of DAP12-NKG2C activating immunoreceptor complex, which has evolved to regulate NK cell activity, ensures that NK cells protect the hosts against pathogens, yet preventing the deleterious NK cell-driven autoimmune responses.

Employing the sequential immunoprecipitation techniques, Feng et al. [10], [11] have characterized the key intramembranous contacts are mediated not by simple one-to-one, charge-paired salt bridges but by a considerably more complex electrostatic network. Recently, the NMR structure of the DAP12-NKG2C TM heterotrimer was determined in tetradecylphosphocholine (TDPC)-SDS mixed micelles. The NMR structure provides the first structural insight into the TM contacts within an assembled DAP12-NKG2C immunoreceptor complex [12]. Nonetheless, this complex structure shows a puzzling aspect that only one of the acidic residues is in the direct contact with the basic residue of the NKG2C. Furthermore, the other Asp residue that does not contact with NKG2C faces to the outside of the hydrophobic core, which may be more energetically favorable for the interaction with the basic residue of another NKG2C. In contrast, experimental data clearly demonstrate there is a strict requirement for a pair of acidic residues for the assembly of the trimer [10], [11]. Moreover, whether the NMR structure of the DAP12-NKG2C TM heterotrimer in micelles represents the TM-TM association within the lipid bilayer remains unknown.

Molecular dynamics (MD) simulation is an important tool for studying the TM-TM association in atomic detail in the lipid bilayer membrane system. With the increasing computer power and the progress in simulation algorithms, it is possible to study the TM-TM association in a well-defined lipid bilayer environment by using MD simulations. Atomistic MD simulations have been used to investigate the self-assembly of glycophorin A (GpA) and to estimate the energetics of TM helix dimerization [13], [14]. Systems with longer effective time scale can be simulated by taking advantage of the Coarse-Grained (CG) approach, in which small groups of atoms are treated as single particles [15]–[17]. The NMR structure of DAP12-NKG2C trimer complex was then refined by Cheng et al. [18] in explicit micelle and bilayer membranes through molecular dynamics simulations, showing a globally similar structure but with different side-chain orientations/conformations of 5 required interfacial residues. There are still a lot of structural details missing in their studies, such as the self-assembly dynamics of DAP12-NKG2C trimer and whether the two aspartic acids in DAP12 dimer interact with lysine in NKG2C in the same pattern. In 2013, Sun et al. [19] discussed the possibility/impossibility that DAP12 dimer associates with two NKG2C. However, the expected unstable tetramer complex consisting of two DAP12TM and two NKG2CTM helixes cannot be observed in their simulations. Much longer simulation time or polarizable force field would be needed as they suggested. Therefore, in this study, we combined CG MD and atomistic simulation methods to resolve two basic and crucial questions in DAP12-NKG2C assembly. The first one is to analyze the dynamics and self-assembly of the DAP12-NKG2C TM heterotrimer in the lipid bilayer membrane in detail, improving the understanding of previous experimental observations and simulation results. We also simulated the dynamics of point mutations involving the polar residues which are important for the self-assembly of the DAP12-NKG2C TM heterotrimer. The second one is to exclude the possibility that another NKG2C can stably associate with DAP12-NKG2C heterotrimer. Based on our simulation results, we propose a revised model for the self-assembly of the DAP12-NKG2C activating immunoreceptor complex and a plausible explanation for the fact that only one NKG2C associates with the DAP12 homodimer.

## Materials and Methods

### Coarse-Grained MD Simulation

All CG-MD simulations were performed using GROMACS 4.5.3 (www.gromacs.org) [20]. The MARTINI force field was used for the simulation system, especially MARTINI force field 2.2 for proteins [17], [19], [21], [22]. This force field was improved for the polar interaction. The coordinates for the DAP12 dimer TM and NKG2C TM were obtained from NMR structures PDB: 2L34 and PDB: 2L35 respectively [12]. The assigned backbone particle for glycine (Gly) residues in the TM segments of the helices changed to atom type Na as the Na-Na pairwise interaction is semiattractive as described in the work by Psachoulia et al. [23] on GpA TM homodimerization and Chng et al. [24] on the integrins TM hetero-dimerization. This alteration is to mimic the interaction for GxxxG motif in the helices association, as the Gly backbones are exposed.

For the investigation of the DAP12-NKG2C trimer, an initial DAP12 dimer and NKG2C TM were placed into a preformed 186 DPPC bilayer with a backbone distance of approximately 55 Å; for analysis of the mutational effect, a representative trimer structure was first selected by clustering analysis and subsequently modified to all mutants using Modeller [25], inserting into the center of the DPPC bilayer at last; to study the possibility of tetramer formation, the representative trimer structure, along with an additional NKG2C TM, were separately placed into the DPPC bilayer at a distance of 55 Å. The N- and C-terminal of the peptide were not acetylated or amidated. Ions were added to electrically neutralize the system. The sequences of DAP12, NKG2C, and their mutants are listed in Table 1; all simulation systems are listed in Table 2.

**Table 1:** TM Sequences of DAP12, NKG2C, and their mutants.

TM Helix	TM sequence
DAP12	VLAGIVMGDLVLTVLIALAVY^62^
D50L	VLAGIVMG**L**LVLTVLIALAVY
D50N	VLAGIVMG**N**LVLTVLIALAVY
T54A NKG2C K52L	VLAGIVMGDLVL**A**VLIALAVY GIISIVLVATVLKTIVLIPFL^6^ GIISIVLVATVL**L**TIVLIPFL

**Table 2:** Summary of Simulations.

Type	Components	Simulation Setup
CG	DAP12-NKG2C-DAP12 +186 DPPC	3000 ns×3
CG	DAP12-NKG2C-D50L +186 DPPC	3000 ns×3
CG	DAP12-NKG2C-D50N +186 DPPC	3000 ns×3
CG	T54A-NKG2C-T54A +186 DPPC	3000 ns×3
CG	DAP12-K52L-DAP12 +186 DPPC	3000 ns×3
AT	DAP12-NKG2C-DAP12 +128 DPPC	50 ns×3
CG	DAP12-NKG2C-DAP12 + NKG2C+186 DPPC	3000 ns×3

Lennard-Jones interactions were shifted to zero between 9 and 12 Å, and electrostatics were shifted to zero between 0 and 12 Å, with a relative dielectric constant of 15. The non-bonded neighbor list was updated every 10 steps with a cutoff of 14 Å. A Berendsen thermostat [26] was used for temperature (323 K, coupling constant 1 ps) and pressure (1 bar, coupling constant 5 ps, compressibility 4.5×10^−5^ bar^−1^, semiisotropic) coupling. The integration step was 20 fs. These parameters follow from the recommendations of Marrink et al. [27]. The energy of each system was repeatedly minimized and then position-restrained in a 3 ns simulation to allow for better packing of the lipid molecules around the TM helices. Though well repeatability was showed with this simulation method in our previous paper studied on the dimerization of DAP12, we still apply a large sample amount (more than 10 times) in consideration of sample stability. Each CG system started with the same initial configuration, but with different initial velocities. The results were in great accordance (data not shown). Therefore three 3 µs production simulations were shown for the trimer-WT and mutants in this paper for clarity.

### From CG to Atomistic Representation

The reconstruction of a CG model to atomistic (AT) model was done in two steps as previous studies described [28], [29]. Briefly, the representative DAP12-NKG2C trimer structure was clustered from CG-MD trajectories and converted to an AT model by Pulchra software [30] initially; secondly, this AT model was inserted into a pre-equilibrated atomistic model of lipid bilayer using the InflateGRO method [31]. The system was subjected to 1000 steps of steepest descent energy minimization and 1 ns of molecular dynamics (MD) using a weak position restraint (103 kJ mol^−1^ nm^−2^) on the non-hydrogen protein atoms.

### Atomistic MD Simulations

Atomistic MD simulations were performed using the GROMOS96 53a6 force field [32] after the conversion to atomistic representation. The GROMOS96 53a6 force field was proved to be practicable in quite a range of recent studies on transmembrane proteins in bilayers [14], [33]–[35]. The united-atom lipid models generated for GROMOS96 53a6 force field was used [36]. Water molecules were described using the SPC model [37]. The Berendsen thermostat (323K, coupling constant 0.1 ps) and Parrinello-Rahman barostat (1 bar, coupling constant 2 ps, compressibility 4.5×10^−5^ bar^−1^, semiisotropic) were utilized for temperature and pressure coupling. LINCS algorithm [38] was employed to constrain bond lengths. Long-range electrostatic was modeled up to 9 Å using the Ewald particle mesh. The 14 Å cutoff distance was used for van der Waals interactions. Every system was energy minimized using the conjugant gradient method and subsequently equilibrated with protein Cα atoms harmonically restrained for 0.5 ns (force constant = 1000 kJ/mol/A2). Production simulations for 50 ns were performed three times.

All trajectory analyses were computed using GROMACS tools. Visualization and graphics were performed with VMD software [39].

## Results

### Coarse-Grained Simulation of Self-Assembly of DAP12-NKG2C TM Heterotrimer

**Figure 1:**
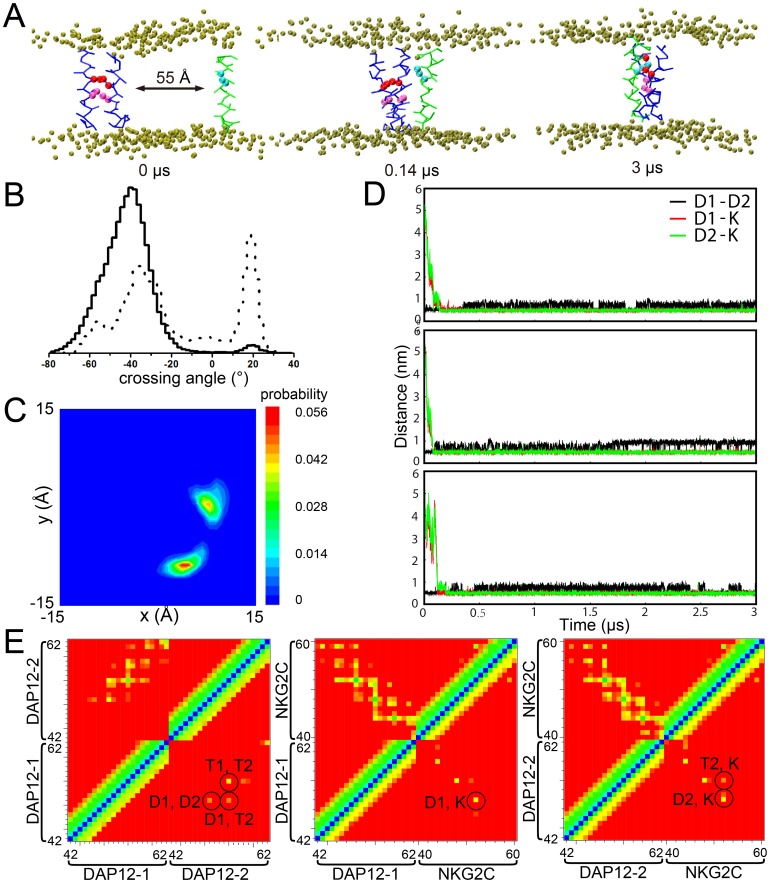
CG-MD simulation of DAP12-NKG2C-DAP12 trimer. A. The initial system configuration consists of the DAP12 dimer (blue) and NKG2C TM (green) in a DPPC bilayer (tan) separated by ∼55 Å. During the 3 us CG-WT simulations, they interact with each other and form a hetertrimer. B. Crossing angle histograms of DAP12 dimer in dimer formation (dashed line) and in trimer formation (solid line). C. Spatial distribution of the DPA12 dimer in the reference frame of NKG2C TM. Blue and red indicate low and high probabilities of finding two DAP12 helices at that position. D. The evolution of minimum distance between side chain beads of Asp1 and Asp2 (black), Lys and Asp1 (red) and Lys and Asp2 (green). E. Interhelical contact matrices for the trimer simulation ensemble calculated using a 10 Å (upper half) and 7 Å (lower half) distance cutoffs. The colors indicate the distances (all particles) of the contacts ranging from 0 Å (blue) to 10 or 7 Å (red). For the lower half of the matrix key interhelical contacts are circled.

The initial structures of the DAP12 TM dimer (PDB: 2L34) and the NKG2C TM (PDB: 2L35) were obtained from the NMR structure, and subsequently placed into a preformed DPPC bilayer with a backbone distance of approximately 55 Å between the dimer and NKG2C. Such a significant separation between the helices ruled out an effect of inter-helix interactions at the beginning of the simulation, as the distance was much larger than the cutoff distance for electrostatic and van der Waals interactions. Three independent simulations were run for 3 µs with different sets of starting velocities. During the simulations, the DAP12 TM dimer and the NKG2C TM drifted through the DPPC bilayer randomly and eventually assembled. As shown in Fig. 1A, the DAP12-NKG2C trimer was formed spontaneously within a few hundred nanoseconds and lasted until the end of the simulation. Under the self-assembly of the trimer, both aspartic acid (Asp) and threonine (Thr) residues which form the dimerization interface of the DAP12 TM dimer [40], turned toward the lysine (Lys) of NKG2C TM. We found the interaction between positively charged Lys and negatively charged Asp pair in the trimer, which lasted throughout the simulations.

Another notable event that occurred during the simulations was the crossing angle of DAP12 dimer changed from approximately 20° to about −40°(Fig. 1B), which meant the conformation of the dimer converted from a left-handed (LH) to a right-handed (RH) packing model. To further investigate the packing of DAP12-NKG2C trimer helix, the spatial distribution of DAP12 TM chains was calculated using backbone particles of NKG2C TM as a reference system. As Fig. 1C depicted, the distribution of each DAP12 chain concentrated on a single point. It is also demonstrated that the spatial distributions of each DAP12 TM chain were on a circumference in the reference frame of NKG2C. Furthermore, the formation of stable heterotrimer was assessed by monitoring the minimum distances between Lys of NKG2C and Asps of the DAP12 dimer, as a function of time. As shown in Fig. 1D, when NKG2C TM stayed in close proximity to the dimer interface of DAP12 TM to form a stable trimer, the Asps of DAP12 dimer slightly moved away from one another. The distances between Lys of NKG2C and Asp of DAP12 were shorter than the distance of two Asps of the DAP12 dimer, which indicated both Asps can form stable salt bridges with Lys after self-assembly of DAP12-NKG2C trimer. More interestingly, the distances between each DAP12 Asp and NKG2C Lys were as close as about 0.5 nm, which meant both Asps in DAP12 contribute equally to the stable trimer formation.

To access the key interactions involved in heterotrimer assembly, the inter-helical contacts were examined in greater depth by calculating the inter-helix distance matrices averaged over all three simulations (Fig. 1E). It was evident that the interactions between DAP12-1 and DAP12-2 are dominated by key residues of Asp and Thr, while the interactions between each DAP12 TM and NKG2C TM are both dominated by the Asp and Lys pair. Moreover, another notable finding was Thr in DAP12 TM can be involved in both interhelical interaction with Asp and interhelical interaction with Lys (circled in Fig. 1E).

### Atomistic Simulation of DAP12-NKG2C TM Trimer

To fully assess and refine the conformational dynamics and stability of the DAP12-NKG2C trimer model generated by the CG self-assembly simulations, and more importantly, to evaluate the electrostatic network among polar interactions, we performed three independent simulations of a 50 ns duration for WT trimer structures in a full atomistic representation. The initial structure was generated from CG simulation results, using the CG2AT fragment-based procedure.

**Figure 2:**
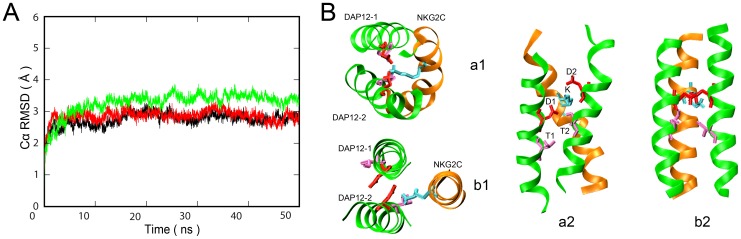
AT-MD simulation of DAP12-NKG2C-DAP12 trimer. A. Time dependence of backbone RMSD for DAP12-NKG2C-DAP12 trimer of three samples. B. Top and side view of the interaction between the key interfacial residues in DAP12-NKG2C-DAP12 complex in PDB structure: 2L35 (b1, b2) and in our DPPC bilayer system (a1, a2) respectively. Polar residues Asps (red) and Thrs (pink) in DAP12 dimer and Lys (cyan) in the NKG2C TM are labeled. All other side chains are omitted for clarity.

**Figure 3:**
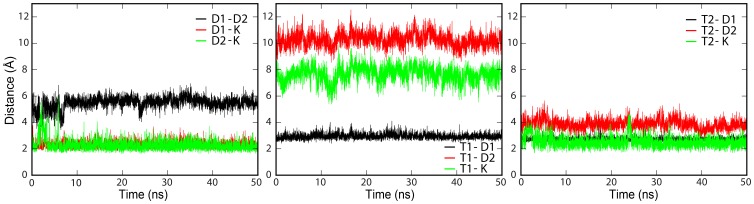
Distance between the key residues versus time in AT-MD simulation of DAP12-NKG2C-DAP12 trimer.

The Cα root mean-squared deviations (RMSD) from the initial structure against simulation time were calculated to assess the conformational stability of the trimer structure. As Fig. 2A shown, the Cα RMSD for the trimer in all simulations reached a plateau rapidly, indicating that the stable and equilibrated trimer structures were obtained. Simulations with longer time scale, 200 ns, were also performed. Plateau was reached within similar short time in the Cα RMSD analysis (data not shown), indicating that 50 ns simulations was long enough to obtain a representative structure in consideration of time consuming. Cluster Method was applied to analyze the conformations of the trimer, as well as the representative structure (Fig. 2B) obtained from all three simulations. In our representative structure, crossing angles of two DAP12 helices were stable and obviously larger than those in NMR structure (Fig. S1 in File S1), indicating these three helices prefer to form a tight packing to lower the system energy by successfully burying polar residues within the TM hydrophobic core. More interestingly, two Asps of the DAP12 dimer faced up and down to Lys of NKG2C respectively for facilitating the formation of salt bridges. These results showed some differences with the available NMR structure, in which all three polar residues (two Asps and Lys) remained the same plane, while one Asp faced out to the hydrophobic core, which is energetically unfavorable. In addition, the Thr in DAP12-2 (Thr2) was found to have a high probability of forming hydrogen bonds with Asp (Asp1) in the other DAP12 chain (DAP12-1) and Lys in NKG2C. Stable salt bridges between Asp1-Lys and Asp2-Lys, as well as hydrogen bonds between Thr2-Asp1 and Thr2-Lys, were further confirmed from the distances between residues in all three trajectories in perfect accord. Thus, we only show details of one sample in Fig. 3, and the other two samples are shown in Fig. S2 in File S1.

The structure of DAP12-NKG2C trimer in our AT MD simulations showed quite a great stability along with all the analysis of Cα RMSD, crossing angle, and residue contacts. Then we come to a conclusion that the DAP12-NKG2C trimer not only decreased the repulsive interaction between Asp residues, but also increased the attractive interaction between Asps and Lys, by adopting this packing pattern to lower the system energy. Beside the strong polar interaction, Thr-linked intrahelical and interhelical hydrogen bonds also made contributions to the stability of the trimer conformation. Those results were in great agreement with recent studies by Cheng et al. [13] and Sun et al. [14], and give more information about the interaction mechanism of DAP12-NKG2C trimer in details. In this sense, the association of DAP12 dimer and NKG2C stabilized the dimer, for a signaling purpose.

### Mutational Effect of DAP12-NKG2C Heterotrimer

**Figure 4:**
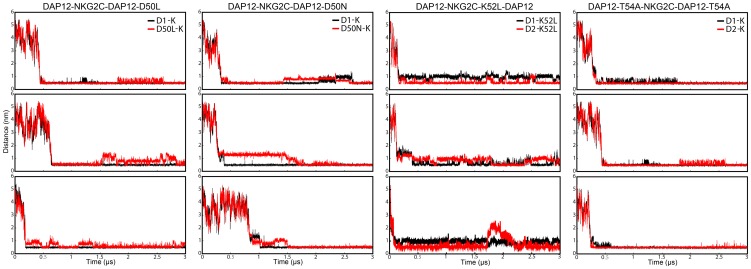
The evolution of minimum distance between side chain beads of Asp1 (or residues Asp1 mutated to) and Lys (or residue Lys mutated to) (black), Asp2 (or residues Asp2 mutated to) and Lys (or residue Lys mutated to) (red) of all four mutants of the trimer: DAP12-NKG2C-D50L, DAP12-NKG2C-D50N, DAP12-K52L-DAP12, and T54A-NKG2C-T54A.

Previous experimental studies have shown the side chains of charged polar residues are strictly essential for assembly of DAP12-NKG2C heterotrimer, while mutating polar residues leads to significant reduction in trimer assembly. To confirm the importance of polar residues in trimer assembly, we explored the structure and conformational stability of 4 mutants (DAP12-NKG2C-D50L, DAP12-NKG2C-D50N, T54A-NKG2C-T54A, and DAP12-K52L-DAP12) via CG simulation. Minimum distances between Asps in DAP12 and Lys in NKG2C were plotted as a function of time in Fig. 4. The final conformations of mutants, however, differed from the wild type trimer simulations and from one another in the extent, to which subsequent dissociation of the trimer occurred in each simulation. In particular, DAP12-NKG2C-D50N mutant, which was proven to maintain the dimerization state of DAP12 in our previous study, affected the stability of trimer dramatically. Thus, a conclusion was deduced that both Asps in DAP12 dimer were crucial to the trimer formation. Noticeably, the DAP12-K52L-DAP12 mutant totally disrupted the polar interaction in the trimer, while DAP12 dimer maintained its conformation with more proportion of a left-handed packing model due to the steric hindrance of the receptor (Fig. S3 in File S1). It is interesting that mutating both Thrs to Alas in DAP12 dimer did have an effect on the stability of the interaction between Asp and Lys as shown in Fig. 4, indicating the function of Thrs in DAP12 dimer is to help stabilize the tightly packing formation of the DAP12-NKG2C trimer. The modest effect was in great accordance with the role of Thrs played in the association of DAP12-NKG2C. Spatial distributions of the mutants were analyzed in Fig. S4 in File S1, and disruptive effect was also found. These findings were in agreement with previous mutagenesis studies, and prove the importance of a five-polar-residue motif in DAP12 assembling with its receptor from a molecular structural aspect.

### Stable Trimer Rules, Not Tetramer

**Figure 5:**
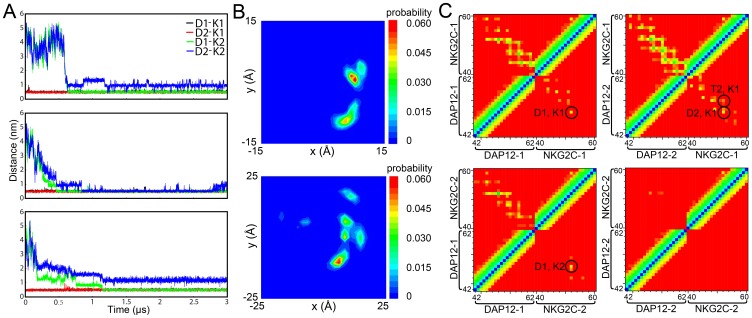
CG simulation of DAP12-NKG2C-DAP12 trimer with an additional NKG2C receptor. A. The evolution of minimum distance between side chain beads of Asp and Lys. B. Spatial distribution of the DAP12 dimer in the reference frame of NKG2C-1 (upper panel) and NKG2C-2 (lower panel). C. Interhelical contact matrices of DAP12-NKG2C-1-DAP12 and DAP12-NKG2C-2-DAP12.

Until now, an open question still remained to be answered: Is there any possibility another NKG2C receptor could associate with the stable DAP12-NKG2C heterotrimer to form a stable tetramer? To answer this question, the representative trimer structure obtained in the CG simulations and another NKG2C TM (NKG2C-2) were selected to be set into the DPPC box separately as a starting system, following simulations that were run for 3 µs duration. It was quite interesting that NKG2C-2 TM, as well as NKG2C-1 TM, is located at the same side of the DAP12 dimer in all three samples, but not the opposite side. As shown in Fig. 5A, it approximately took 1 µs for NKG2C-2 TM to approach the stable DAP12-NKG2C trimer. The side chain of Lys in NKG2C-2 (Lys-2) was toward Asp-1 of the DAP12 dimer, and remained at a distance from Asp-2. Evolution of TM backbone distance between DAP12 and NKG2C (data not shown) indicated both chains of the DAP12 dimer had a stable interaction with the original NKG2C-1 in the heterotrimer throughout the simulations, instead of NKG2C-2 which was about 10 Å away from DAP12 dimer. This phenomenon was further proven by the analysis of residue distances between each Asp and Lys (Fig. 5A). To further prove the disability of the tetramer, spatial distribution and residue contact were examined. The spatial distributions of the DAP12 dimer respectively referred to NKG2C-1 and NKG2C-2 were essentially different (Fig. 5B). In the NKG2C-1 frame, each DAP12 had only one probability density maxima within the distance range of 1.5 nm; while in NKG2C-2 frame, the distribution was quite disordered and the distance range was enlarged to 2.5 nm for a better view. Residue contacts mapped in Fig. 5C demonstrated NKG2C-1 and both chains of DAP12 dimer still maintained the same contact interface dominated by residue Asps, Thrs, and Lys; whereas NKG2C-2 had minute contact with DAP12-1, and no contact with DAP12-2, in a 10 Å distance cutoff. In conclusion, we firstly got the solid simulation results to explain why DAP12-NKG2C trimer cannot form a stable tetramer with another NKG2C, thus proving the DAP12 dimer can only associate with one NKG2C.

## Discussion

The ligand-binding and signal-transducing modules of most activating immunoreceptors are separately synthesized and assembled into various membranes, followed by association into activating immunoreceptor complex through the electrostatic network among their transmembrane α-helices. Amongst such, the DAP12-NKG2C activating immunoreptor complex is an interesting model to study assembly of a diverse group of receptors with their dimeric signaling modules. Since the NMR structure of DAP12-NKG2C trimer was revealed, more structural insight led researchers to focus on the assembly and dynamics mechanism. In this paper, we sought to understand how this particular electrostatic network among the polar residues creates a stable and specific contact site deep in the lipid bilayer that directs the assembly of DAP12-NKG2C activating immunoreceptor complex. Furthermore, the molecular simulation results are expected to provide a clear explanation that DAP12 dimer can only associate with one NKG2C receptor at one time.

As to provide simulations with a longer time scale compared to the studies done by Sun et al. and Cheng et al., coarse grained methods was applied to study the assembly dynamics between DAP12 dimer and NKG2C. The results of the CG-MD study of DAP12-NKG2C trimer showed the NKG2C and DAP12 dimer quickly formed a trimer and kept stable throughout the simulations. Examining greater detail of the complex structure from AT-MD simulations, the resulting structure of DAP12-NKG2C trimer showed significant similarities and differences with the available NMR structure. Instead of facing the membrane hydrophobic core in NMR structure (PDB: 2L35) which is energetically unfavorable, both side chains of Asps in DAP12 oriented toward the interface of the trimer complex. To decrease electrostatic repulsion between both Asps of the DAP12 dimer, both chains of DAP12 dimer shifted away and moved slightly vertical to facilitate the formation of two salt bridges between both Asps and Lys. These two stable salt bridges were further proven from the distances between each DAP12 Asp and NKG2C Lys, which were equally as close to approximately 0.5 nm and lower than the distance between both Asps of the DAP12 dimer. Aside from the critical aspartic acid residue, the most striking effect of DAP12TM residue on the receptor assembly was Thr. In our simulation, Thr-2 was found to have a high probability of forming hydrogen bonds with Asp-1 in the DAP12-1 chain and Lys in NKG2C. Stable salt bridges between Asp-1 and Lys; as well as Asp-2 and Lys, hydrogen bonds between Thr-2 and Asp-1, Thr-2 and Lys were further confirmed from: 1) examinations toward distances between key residues, 2) spatial distribution, and 3) residue contact between TMs. Additionally, assembly of DAP12 dimer revealed a bimodal crossing angle distribution and switched between a left-handed and right-handed packing model [40]. After association with NKG2C, the DAP12 dimer was firstly found to adopted a −40° right-handed packing pattern as shown in Fig. 1. These results provide a clue to explain how the receptor helps to stabilize the conformation of DAP12 dimer during the assembly of DAP12-NKG2C trimer.

As the mechanism of the DAP12-NKG2C trimer was unveiled, the mutational effect toward key residues Asp, Thr, and Lys in the trimer was studied by using CG-MD simulations. The results were in accordance with previous experimental data. The stable trimer could not be maintained if one Asp was mutated to either leucine (Leu) or the most conservative asparagine (Asn), which indicated both Asps in the DAP12 dimer were crucial to trimer formation with its receptor. Even when mutating Thr to Ala, the polar interaction between Asp and Lys are affected modestly. This effect directly identified the importance of Thr in DAP12-NKG2C trimer formation. NKG2C could not associate with the DAP12 dimer when Lys was mutated to Leu, while DAP12 dimer maintained dimerization. These results further proved the five-polar-residue motif was crucial to association of the trimer.

Finally, another NKG2C was added to our trimer system to answer the critical question of why only one NKG2C could associate with the DAP12 dimer. It took a relatively longer time for NKG2C-2 to get close to DAP12 dimer, as the Asp pair was already occupied by Lys in NKG2C-1. More interestingly, NKG2C-2 always moved to the same side as NKG2C-1. Residue distances between Asps and Lys-1, Asps and Lys-2 revealed that NKG2C neither have stable polar interaction with the DAP12 dimer nor destroyed the association among the original trimer. Furthermore, spatial distribution analysis showed the DAP12 dimer and NKG2C-1 shared the same interface as our trimer investigation described, while DAP12 dimer kept a relatively long distance from NKG2C-2 and the spatial distribution seemed to be chaotic. The same conclusion was also obtained from residue contact analysis. All the results indicated the assembly mechanism of DAP12-NKG2C trimer ruled out the possibility of tetramer formation with an additional NKG2C.

**Figure 6:**
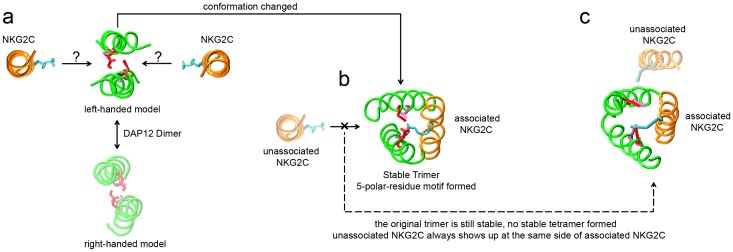
Proposed model for DAP12-NKG2C complex assembly. (a), (b), and (c) are three different phases of DAP12-NKG2C complex following the unique assembly principle. The structure in (c) was obtained from our CG study, and then we converted it to an atomistic model for a better view. Polar residues Asps (red) and Thrs (pink) in DAP12 dimer and Lys (cyan) in the NKG2C TM are labeled.

Based on the results from this investigation, we propose a new model for the assembly of TM domain of DAP12-NKG2C activating immunoreceptor complex in Fig. 6. The DAP12 dimer originally has two possible binding sites according to the symmetrical structure. Once receptor NKG2C with an available binding site has associated with the DAP12 dimer, strong polar interactions and hydrogen bonds drive rotational and vertical movement of the DAP12 dimer to form a stable DAP12-NKG2C trimer. The electronegativity of the DAP12 dimer redistributes and a newly balanced electrostatic network is rebuilt. Once the assembly of the trimer has formed, there is no available binding site on the other side of the DAP12 dimer for another NKG2C. These notions broaden our horizon to understand the organizing principle of a host of activating immune receptors, as our simulation system can be further applied to various other studies involving polar interactions in a bilayer system.

In summary, we gained greater insight in the mechanism of TM interaction in DAP12-NKG2C trimer, and confirmed the important role of each residue in a five-polar-residue motif including 2 Asp, 2 Thr, and 1 Lys in both CG and AT MD simulations. Furthermore, we provide clear evidence to support the conclusion that the DAP12 dimer can associate with only one NKG2C receptor at one time.

## Supporting Information

File S1 (PDF)
