# Supplementary material for: Molecular Dynamic Simulation of the Self-Assembly of DAP12-NKG2C Activating Immunoreceptor Complex
Source: PLoS One. 2014 Aug 22;9(8):e105560. doi: 10.1371/journal.pone.0105560 (PMC4141757; doi:10.1371/journal.pone.0105560)
Supplement: File S1 — (PDF) [file pone.0105560.s001.pdf]

Supporting Information

Molecular Dynamic Simulation of the Self-Assembly of DAP12-NKG2C Activating Immunoreceptor Complex

Peng Wei[†], Li-Da Xu[†], Cheng-Dong Li, Fu-De Sun, Long Chen, Tian-Wei Tan, Shi-Zhong Luo[*]

[*]Beijing Key Laboratory of Bioprocess, College of Life Science and Technology, Beijing University of Chemical Technology,
Beijing 100029, P.R.China

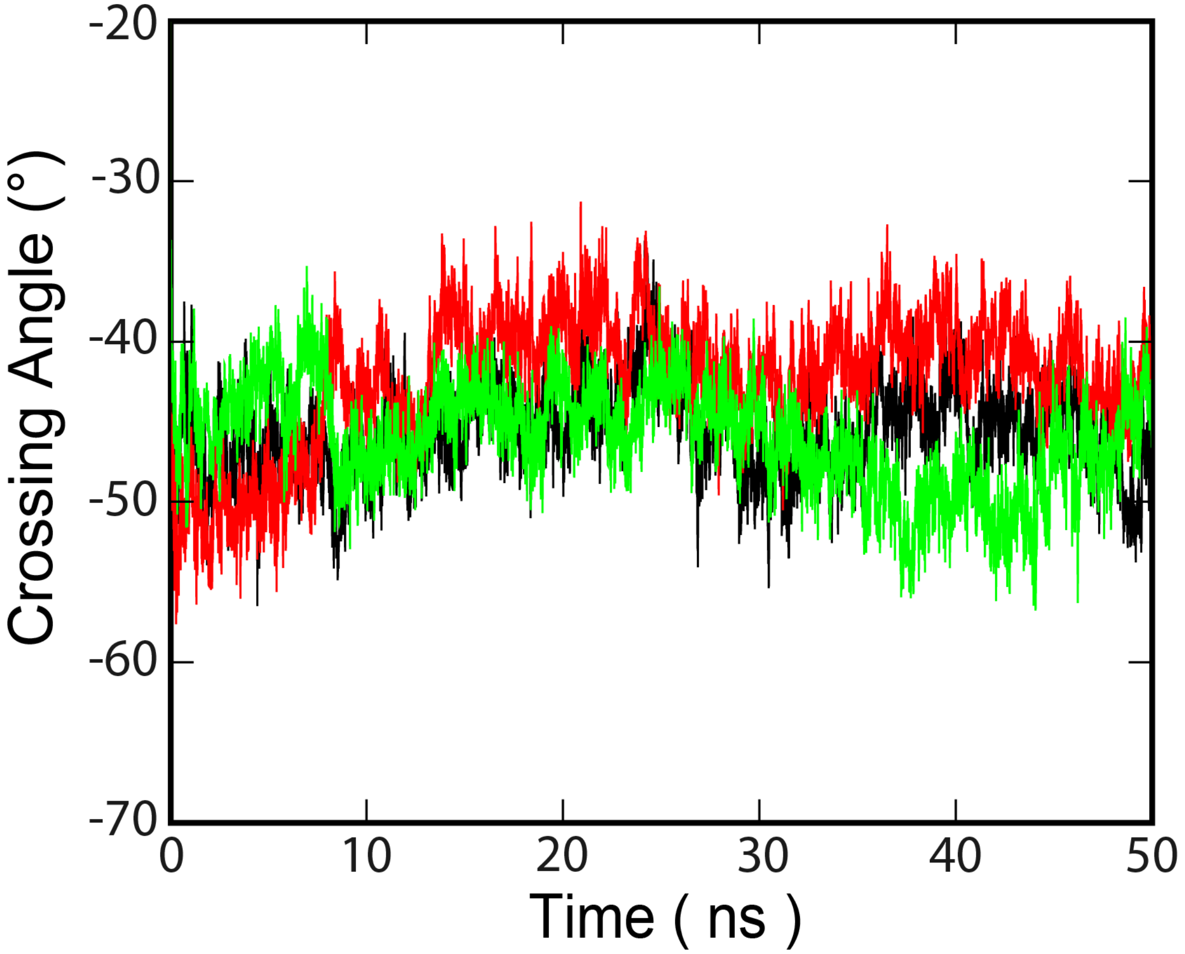

Figure S1: Crossing angle analysis based on AT-MD simulation of DAP12-NKG2C-DAP12 trimer. The figure shows the evolution of crossing angles of the DAP12 dimer. The analysis is carried out on all the three simulations (black, red, and green).

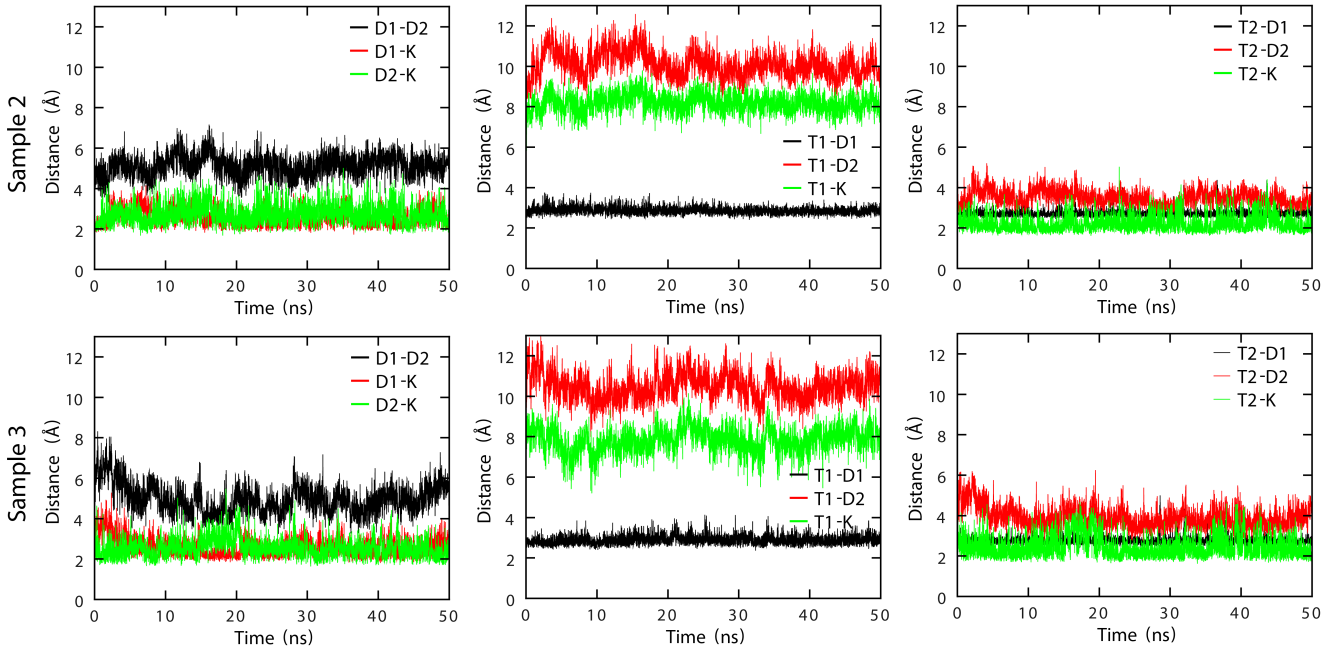

Figure S2: Distance between the key residues versus time in AT-MD simulation of DAP12-NKG2C-DAP12 trimer. Sample 2 and Sample 3 were shown; and Sample 1 was shown in Figure 3.

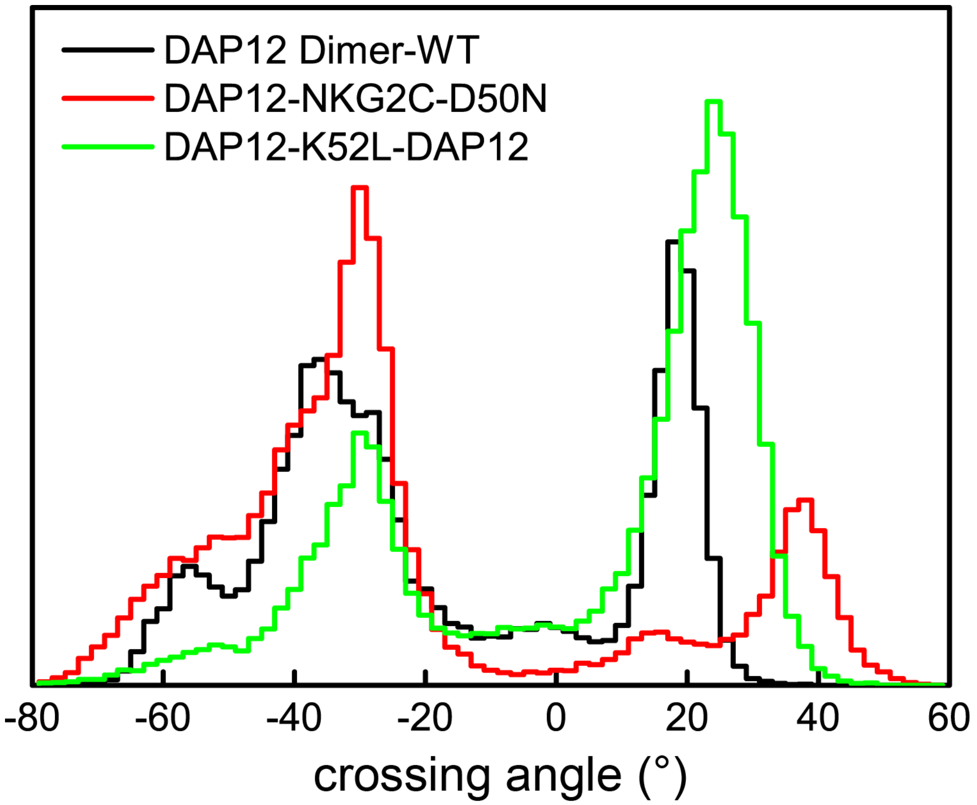

Figure S3: Convergence analysis of CG-MD simulations based on crossing angle analysis. The figure shows the distribution of the DAP12 dimer in wild type (black), DAP12-NKG2C-DAP12 (red), and DAP12-K52L-DAP12 (green) crossing angles. The analysis is carried out on all the three simulations.

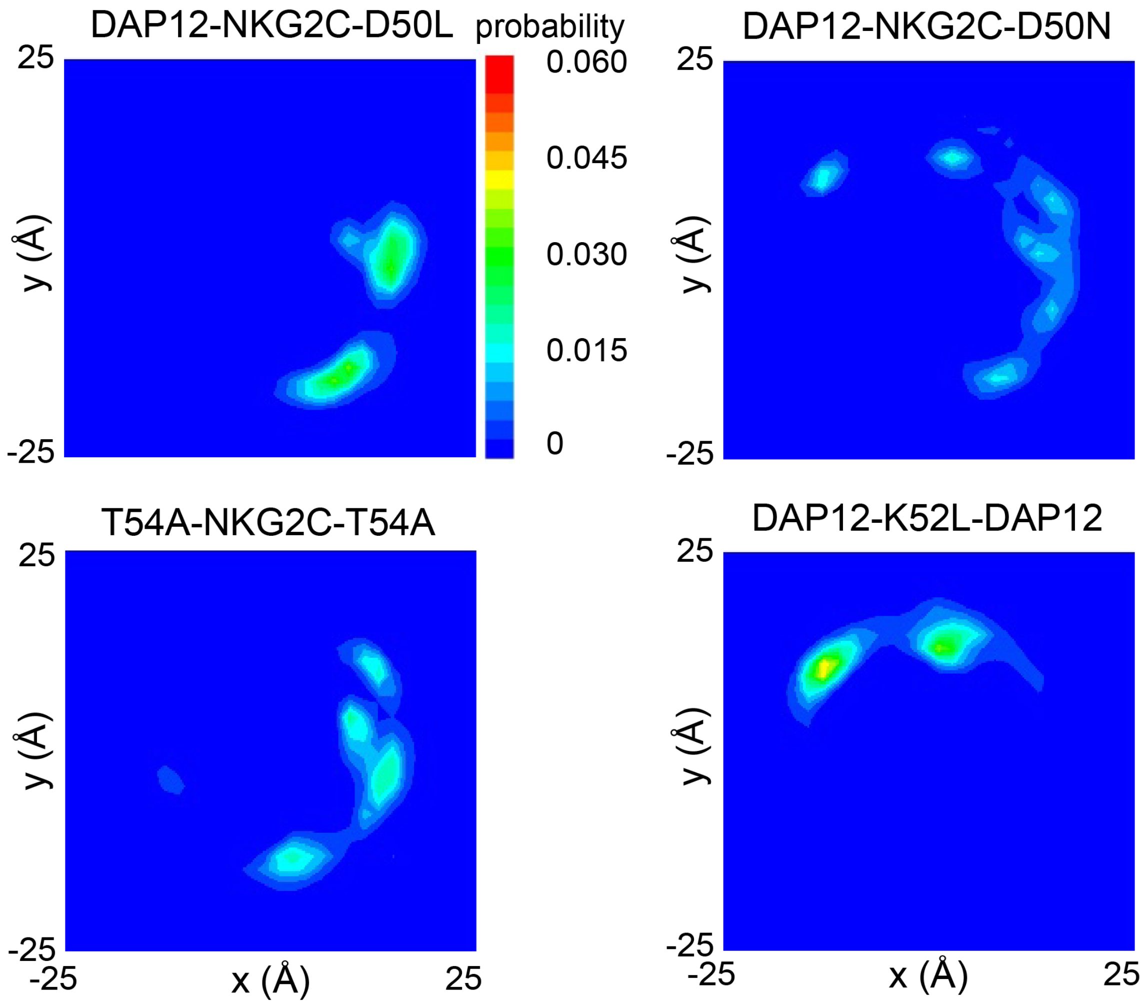

Figure S4: Convergence analysis based on the spatial distribution of the DAP12 dimer in mutants of DAP12-NKG2C-DAP12 trimer with respect to NKG2C. Blue and red indicate low and high probabilities of finding two DAP12 helices at that position.
